# Metabolic modulation and multi-species interaction: *Lactiplantibacillus plantarum*’s impact on *Streptococcus mutans*-*Candida albicans* in a mucosal model

**DOI:** 10.3389/fcimb.2025.1652490

**Published:** 2025-10-08

**Authors:** Ting Li, Yan Wu, Lanxin Zhang, Hiba Alyami, Nora Alomeir, Tongtong Wu, Jin Xiao

**Affiliations:** ^1^ Eastman Institute for Oral Health, University of Rochester Medical Center, Rochester, NY, United States; ^2^ College of Laboratory Medicine, Chongqing Medical University, Chongqing, China; ^3^ Department of Biomedical Informatics, Harvard Medical School, Boston, MA, United States; ^4^ Department of Biostatics and Computational Biology, University of Rochester Medical Center, Rochester, NY, United States

**Keywords:** lactiplantibacillus plantarum, Streptococcus mutans, Candida albicans, galacto-oligosaccharides, oral mucosa, metabolomics

## Abstract

**Introduction:**

*Streptococcus mutans* and *Candida albicans* are key pathogens in dental caries and oral candidiasis. While *Lactiplantibacillus plantarum* and the prebiotic galactooligosaccharides (GOS) have been shown to inhibit these pathogens and their interactions in biofilm and planktonic models, their effects and mechanisms in an oral mucosal context remain unclear.

**Methods:**

This study investigated the impact of *L. plantarum*, alone and in combination with GOS, using an oral mucosal model. The analyses focused on pathogen viability, adhesion, transmigration, virulence expression, mucosal barrier integrity, inflammatory response, extracellular polysaccharide production, and metabolism.

**Results:**

*L. plantarum* reduced the viability of *S. mutans*, inhibited the adhesion of both pathogens to the oral mucosa, and decreased the transmigration of *S. mutans* through the mucosal membrane. It also attenuated the virulence of *C. albicans* by inhibiting hyphae formation and gene expression. Furthermore, *L. plantarum* helped maintain mucosal barrier integrity by mitigating the epithelial inflammatory response induced by the pathogens. The combination of GOS and *L. plantarum* significantly reduced pathogenic extracellular polysaccharide production by S. mutans, creating a metabolic microenvironment less conducive to the survival and interaction of both pathogens. Notably, *L. plantarum* significantly altered the metabolic landscape of these pathogens, especially under GOS conditions.

**Discussion:**

These findings demonstrate that *L. plantarum*, particularly when combined with GOS, exerts inhibitory effects on *S. mutans* and *C. albicans* in an oral mucosal model through metabolic and immunologic regulation. The results highlight the potential of synbiotic strategies (probiotics and prebiotics) for preventing and mitigating oral diseases involving the mucosal barrier and the pathogenesis of *S. mutans* and *C. albicans*.

## Introduction

Early childhood caries (ECC) is the most common chronic disease affecting children worldwide, with a particularly high incidence among disadvantaged preschoolers (Alkhars et al., 2022). This condition results from a significant dysbiosis of the oral environment, primarily due to transmissible pathogens that form virulent biofilms on tooth surfaces (Divaris et al., 2019). The main bacterial agent associated with ECC, *Streptococcus mutans* (*S. mutans*), interacts synergistically with the fungal species *Candida albicans* (*C. albicans*) in both saliva and biofilm environments (Liu et al., 2023). Research has shown that exoenzymes produced by *S. mutans*, especially glucosyltransferase B (GtfB), can bind to *C. albicans*, promoting the synthesis of extracellular glucans and enhancing adhesion and biofilm formation (Hwang et al., 2017; Wan et al., 2021). These interactions accelerate microbial colonization and exopolysaccharide (EPS) production, significantly contributing to enamel demineralization, particularly in carbohydrate-rich environments (Rocha et al., 2018). In toddlers with severe tooth decay, structured interkingdom assemblages of *S. mutans* and *C. albicans* exhibit properties such as enhanced antimicrobial tolerance, highlighting the complexity and resilience of biofilms formed by these microorganisms (Li et al., 2023).

ECC begins on tooth surfaces due to the accumulation of cariogenic pathogens such as *S. mutans* and *C. albicans* in dental plaque. Traditional perspectives have primarily focused on the stages following tooth eruption in children. However, recent research indicates that *S. mutans* and *C. albicans* exhibit synergistic interactions in the oral cavity of infants even before tooth eruption, with the oral mucosa serving as the initial site of pathogenesis (Berlutti et al., 2010; Lopes et al., 2023). Mucosal surfaces in the oral cavity are crucial barriers against microbial invasion, and their integrity is essential for maintaining oral health (Samiei et al., 2019). When these barriers are compromised due to dysbiosis, poor oral hygiene, or the presence of pathogens like *C. albicans*, the risk of developing ECC increases (Lopes and Lionakis, 2022; Samiei et al., 2019). *C. albicans* can disrupt tight junctions between epithelial cells, increasing tissue permeability and facilitating the colonization of *Streptococcus* (Xu et al., 2016; Ma et al., 2023; Rollenhagen et al., 2021). This interaction creates a vicious cycle where mucosal damage promotes microbial overgrowth, exacerbating tissue damage and caries progression (Sanz et al., 2017). Despite the importance of mucosal health in ECC development, research in this area is insufficient. There is a significant lack of understanding of the specific mechanisms governing these interactions and how mucosal immunity can be leveraged to prevent or mitigate ECC, leaving healthcare providers with limited strategies for preventing and treating ECC.

Understanding these complex interactions and the role of host defenses is vital for developing strategies to prevent and control ECC and other oral mucosal infectious diseases, such as oral candidiasis. Our previous research has shown that *Lactiplantibacillus plantarum* (*L. plantarum*) can significantly inhibit the growth, virulence, and interactions of *S. mutans* and *C. albicans* in both planktonic and biofilm models (Zeng et al., 2022; Bao et al., 2023). *L. plantarum* is a probiotic known for its ability to adhere to epithelial surfaces, produce antimicrobial substances, and modulate immune responses, making it a promising candidate for protecting against oral pathogens and promoting oral health (Wang et al., 2018; Liu et al., 2020; Choi et al., 2018). Galacto-oligosaccharides (GOS) have also been studied for their prebiotic properties and potential inhibitory effects on various pathogens (Estorninos et al., 2022; Zhu et al., 2023; Cai et al., 2022). We revealed GOS could significantly inhibit the growth of *C. albicans* and *S. mutans* and enhance the anti-fungal ability of *L. plantarum* in a planktonic model (Huang et al., 2023). The potential of *L. plantarum* and GOS to modulate harmful microbial interactions presents an opportunity to develop natural and effective strategies for preventing ECC. However, it remains uncertain whether this inhibitory effect extends to the mucosal interface, a critical area of ECC study.

This study investigates the ability of *L. plantarum* to disrupt *S. mutans*–*C. albicans* interactions and protect mucosal surfaces, focusing on its effects under varying sugar conditions including prebiotic sugar resources. By uncovering the mechanisms behind these interactions, we aim to develop innovative preventive and therapeutic strategies that leverage the beneficial properties of *L. plantarum*. These approaches could significantly improve oral health outcomes, particularly in early childhood. Furthermore, our findings may provide broader insights into microbial interactions in other mucosal environments, informing strategies to address diverse mucosal bacterial and fungal infections.

## Materials and methods

### Bacterial strains and starter preparation

*S. mutans UA159*, *C. albicans SC5314*, and *L. plantarum ATCC 14917* were purchased from ATCC and recovered from frozen stock and subsequently inoculated onto the following media: blood agar (TSA with sheep blood, Thermo Scientific™, Waltham, MA, USA, catalog number R01202), YPD agar (BD Difco™, San Jose, CA, USA, catalog number 242720), and MRS agar (BD Difco™, catalog number 288210). After incubation for 48 hours, *S. mutans, C. albicans, and L. plantarum* were transferred to respective growth media: TSBYE broth (3% Tryptic Soy, 0.5% Yeast Extract Broth, BD Bacto™ 286220 and Gibco™ 212750) with 1% glucose, YPD broth (BD Difco™, 242820), and MRS broth (BD Difco™, 288130). The cultures were incubated overnight. The following morning, 0.5 ml of each overnight culture was added to fresh broth and incubated for 3–4 hours until the optical density (OD) reached the target values: 1.0 for *S. mutans*, 0.8 for *C. albicans* and 2.2 for *L. plantarum*). The morning cultures were then serially diluted to prepare starting concentrations for the mucosal models described below.

### Planktonic model

The starting concentrations of *S. mutans* (10^5^ CFU/mL) and *C. albicans* (10^3^ CFU/mL) were selected to simulate a clinical setting with a high risk of caries. The concentrations of *L. plantarum* (10^7^ CFU/mL) was chosen based on previous studies that demonstrated its inhibitory effects on the growth of both *S. mutans* and *C. albicans*. Dual- and multi-species conditions of *S. mutans*, *C. albicans*, and *L. plantarum* were cultivated in 10 mL of TSBYE broth supplemented with either 1% GOS (BOS Science, New York, NY, USA) or 1% glucose for 20 h (5% CO_2_, 37°C). After incubation, the supernatant was collected by centrifugation and stored at -80 °C for untargeted metabolomics analysis.

### TR-146 cell culture and mucosal model

The TR-146 epithelial cell line was obtained from Millipore Sigma. TR-146 cells (100,000 cells per insert, passages 4 and 10) were seeded onto transwell culture plate inserts (VWR, PET membrane, 3 μm pore size) with medium in both chambers. Cultures were incubated at 37 °C with 5% CO_2_. The mucosal model was ready for microorganism inoculation after reaching confluence in 14 days, with a transepithelial electrical resistance of 50 Ohms×cm². Dual- and multi-species conditions of *S. mutans* (10^5^ CFU/mL), *C. albicans* (10^3^ CFU/mL), and *L. plantarum* (10^7^ CFU/mL) were cultivated in DMEM/F12 medium with 1% glucose, 1% sucrose or 1% GOS for 12–24 hours (5% CO_2_, 37 °C).

### Adherence assay

TR-146 cells grown on Transwell inserts were inoculated with dual- and multi-species suspensions of *S. mutans*, *C. albicans*, and *L. plantarum* for 1 hour. The cells were washed three times with PBS to remove non-adherent microbes. Adherent microbes were detached by scraping with a sterile spatula, suspended in 1 mL of sterile 0.9% sodium chloride solution, and transferred to an Eppendorf tube. The suspensions were serially diluted and plated onto blood agar and YPD agar plates to determine viable counts of *S. mutans* and *C. albicans*, respectively.

### Transmigration assay

Transwell inserts containing TR-146 cells were inoculated in parallel experiments with 10^3^ CFU of *C. albicans* and 10^5^ CFU of *S. mutans*, then incubated at 37 °C for 24 hours. After incubation, the culture medium from the lower chamber was collected, serially diluted, and plated onto selective agar as described above to determine viable cell counts.

### Transepithelial electronic resistance

The barrier function of the TR-146 mucosal model was assessed 24 hours post-infection. TEER measurements were conducted using an Endohm chamber linked to an EVOM epithelial voltometer (World Precision Instruments, Sarasota County, FL, USA). Inserts were placed in the Endohm chamber, and an electrode cap was positioned on top to obtain the TEER reading. Relative TEER in Ω × cm² was calculated by subtracting the background resistance (from an insert with only media) from the average TEER reading per well and multiplying the result by the insert’s surface area (1.12 cm²).

### Quantitative real-time polymerase chain reaction

At 18 hours, 1 mL of culture suspensions was collected from the insert of the mucosal model for RNA extraction. Complementary DNA (cDNA) was synthesized from 0.2 µg of purified RNA using the Bio-Rad iScript cDNA synthesis kit (Bio-Rad Laboratories, Inc., Hercules, CA, USA). Amplification of cDNA and negative controls was performed with SYBR Green Master Mix and a QuantStudio Real-Time PCR System (Thermo Fisher Scientific, Wilmington, DE, USA). Each 20 µL reaction contained cDNA, 10 µM of each primer, and a 2× SYBR Green mix (which includes SYBR Green and Taq DNA Polymerase). The internal reference genes used were gyrA for *S. mutans* and ACT1 for *C. albicans.* Gene expression was quantified using the comparative 2−ΔΔCT method. The sequences of the primers are detailed in **Supplementary Table S1**.

### Transmission electron microscope

The culture media were removed from the transwells and immediately replaced with EM fixative solution containing 2.5% glutaraldehyde and 4% paraformaldehyde in buffer. Samples were fixed at room temperature for 1 hour, rinsed, and post-fixed in 1% osmium tetroxide. Samples were dehydrated through a graded ethanol series, embedded in epoxy resin, and polymerized. Semi-thin (1 µm) toluidine blue–stained sections were used to identify regions of interest, after which ultrathin sections (~70 nm) were cut with an ultramicrotome, and stained with uranyl acetate and lead citrate. Sections were imaged using a transmission electron microscope.

### Immune markers measurement

After 24 hours, the culture suspension was collected from the upper chamber of the mucosal model. The suspension was then centrifuged at 5,000 × g for 10 minutes to obtain the supernatant samples. These samples were analyzed using a multiplex assay to measure various cytokines and inflammatory mediators. The assay was performed according to the manufacturer’s instructions for the MILLIPLEX^®^ Cytokine/Chemokine Magnetic Bead Panel. The analytes measured included Fractalkine, GM-CSF, IFN-γ, IL-1a, IL-1B, IL-1RA, IL-6, IL-8, IL-18, MIP-1a, RANTES, TNFα, ENA-78 and MIP-3a. Results were obtained using a Luminex^®^ 200™ instrument (Luminex, Austin, TX, USA) and reported based on standard curve values.

### Confocal microscopy

Transwell inserts containing dual- and multi-species mucosal models were cultured in a medium supplemented with 1 μM Alexa Fluor 647-labeled dextran conjugate (Invitrogen Corp, Carlsbad, CA) for 12 hours to visualize exopolysaccharides. After this incubation, the inserts were fixed in 4% paraformaldehyde, and Calcofluor White was used to stain the chitin in the cell walls of C. albicans. The membrane was then removed and mounted onto slides using FluorSave reagent. Imaging was conducted using a Leica SP5-AOBS confocal laser scanning microscope (CLSM) attached to a Leica DM I6000 inverted epifluorescence microscope. The acquired images were processed with Imaris v9.5 software (Bitplane AG, Zurich, Switzerland).

### Liquid chromatography-tandem mass spectrometry

Thermo Vanquish HPLC/Orbitrap ID-X MS was used for metabolimics analysis. The samples were thawed on ice and 2 x 100 µL was aliquoted (1 for reversed-phase C18 and 1 for HILIC chromatography) into Eppendorf tubes and extracted with 400 µL of ice-cold methanol. The samples were then vortexed and centrifuged at 18,000 x g at 4 °C for 5 min, A 400 µL aliquot of each supernatant was dried in a 96-well plate under nitrogen at 45 °C and reconstituted in HPLC mobile phases. An aliquot of homogenate was taken to prpare quality control (QC) samples, which were processed in the same manner. The samples were analyzed in 4 separate LC/MS experiments on the Thermo Scientific UHPLC/Orbitrap ID-X mass spectrometer, scanned from m/z 70 to 1000 at a resolution of 120,000. Samples were first run using reversed-phase C18 chromatography (retention of medium to nonpolar metabolites) with electrospray ionization in the positive and negative ionization modes separately on an Orbitrap ID-X mass spectrometer. A separate sample was then analyzed using HILIC chromatography (retention of polar metabolites) also in both ionization modes. Data from the raw outputs were processed using Thermo Scientific’s Compound Discoverer.Metabolomic data processing, analysis, and interpretation using MetaboAnalyst 6.0.

### Statistical analysis

All analyses were performed using SPSS (V24.0). To compare the abundance of *S. mutans*, *C. albicans*, and *L. plantarum* in mucosal models, the CFU/mL values were first converted into natural log values before analysis. Normality tests (Shapiro–Wilk Test) were conducted for converted CFU/mL value, 2^−ΔΔCT and the concentrations of immune markers (concentrations of immune markers converted to natural log value). For normally distributed data, differences were assessed using t-tests for two independent groups and one-way analysis of variance (ANOVA) for multiple group comparisons followed by *post hoc* Tukey’s test. When data were not normally distributed, the Mann–Whitney U test was used to compare the results of the two groups. For LC-MS/MS, Thermo Scientific’s Compound Discoverer was used to extract metabolites, normalize area counts to the pooled QC samples run throughout the LC/MS analysis, identify metabolites via a curated database search, and generate principal component analysis (PCA) plots, fold changes between comparison groups with adjusted p-values. Statistical significance was set at *p* < 0.05. Sample sizes for each experiment are detailed in the figure legends.

## Results

### *L. plantarum* reduces adherence of *S. mutans* and *C. albicans* to oral epithelium

**Figure 1:**
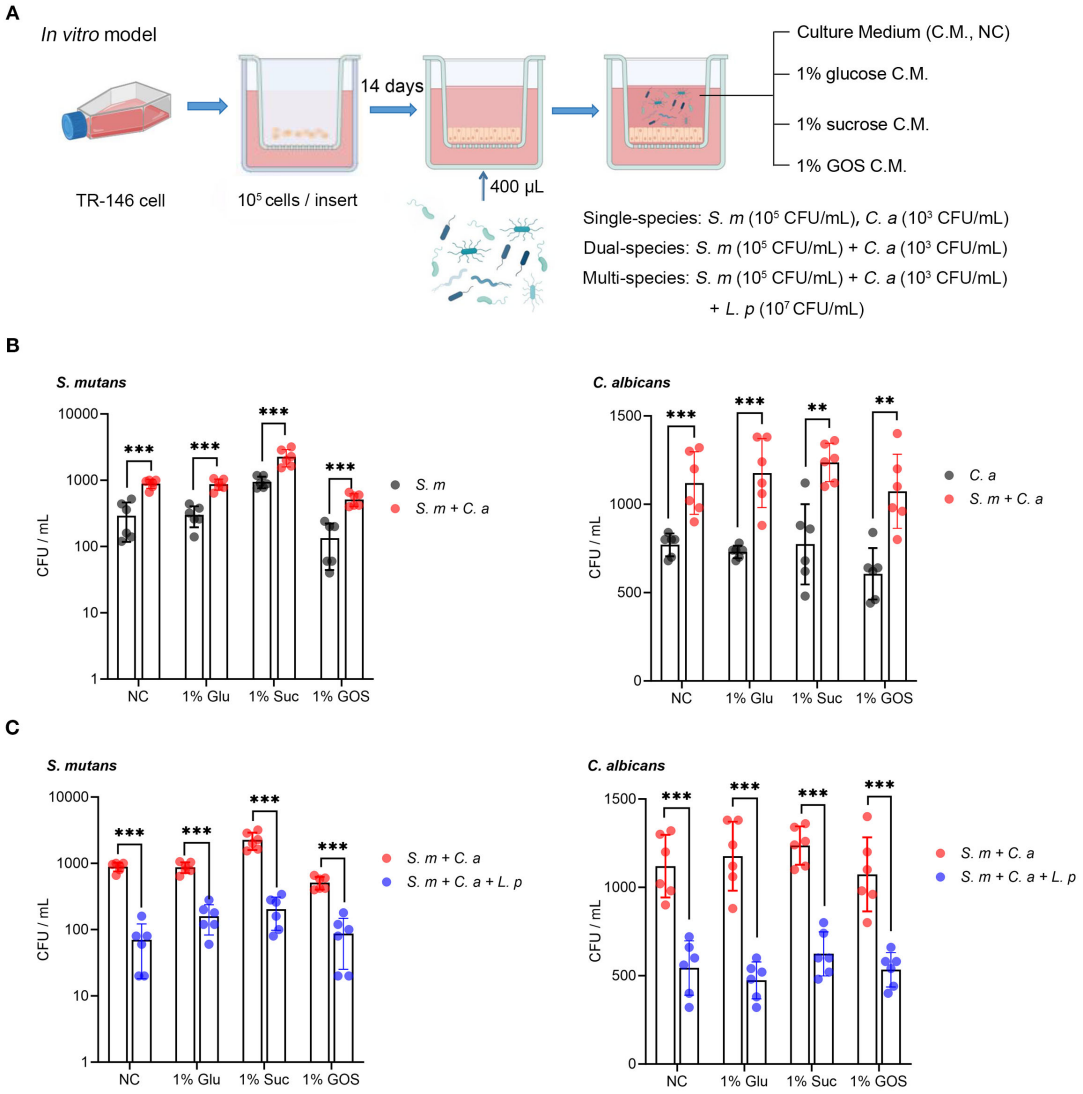
*L. plantarum* reduces adherence of *S. mutans* and *C. albicans* to oral epithelium. **(A)** A schematic model depicting the construction process of a mucosal model infected with different microorganisms (created with BioRender.com). A total of 10^5^ TR-146 cells were seeded onto transwell inserts and cultured for 14 days to establish an *in vitro* mucosal model. Dual- and multi-species conditions with *S. mutans*, *C*. *albicans*, and *L. plantarum* were maintained in DMEM/F12 medium with 1% glucose, 1% sucrose, or 1% GOS (5% CO2, 37 °C), single species as a reference. The multi-species condition was designed to evaluate the effect of *L. plantarum* on the adherence of *S. mutans* and *C. albicans*. **(B)** Adherence of *S. mutans* and *C. albicans* to oral epithelial TR146 cells after 1-hour incubation. **(C)** Reduction in adherence of *S. mutans* and *C. albicans* to oral epithelial TR146 cells after 1-hour incubation. Data are shown as mean ± SD (n = 6). *p*-values were determined by unpaired t test. ** p<0.01, *** p<0.001.

The schematic design of the mucosal model and microbial inoculation (**Figure 1A**). Compared to the single-species model, co-inoculation with *S. mutans* and *C. albicans* significantly increased the adhesion of each species to oral epithelial cells (TR146) after 1-hour incubation in all sugar condition (**Figure 1B**). Notably, the addition of *L. plantarum* to the *S. mutans* and *C. albicans* mucosal model significantly inhibited the adhesion of both species in all sugar-conditions (**Figure 1C**).

### *L. plantarum* inhibits the viability and transmigration of *S. mutans*

**Figure 2:**
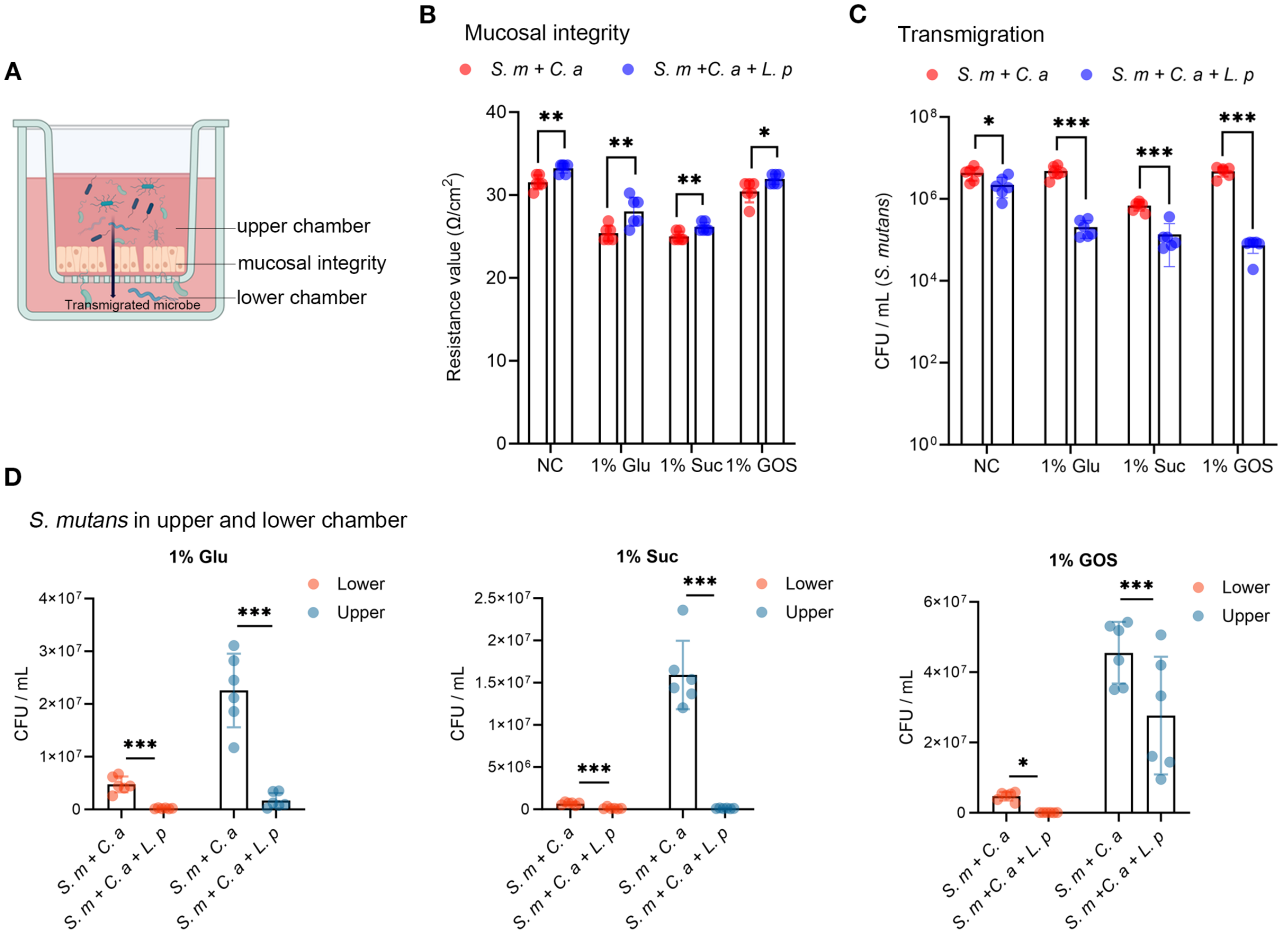
*L. plantarum* inhibits the viability and transmigration of *S. mutans*. **(A)** A schematic model depicting microbial transmigration from upper chamber to lower chamber by disrupting mucosal model integrity (created by BioRender.com). **(B)** TEER values were measured to assess the mucosal barrier integrity. **(C)** Viable counts in colony forming unit (CFU) of *S. mutans* transmigrated from the upper chamber to the lower chamber after 24-hour incubation. **(D)** Viable counts of *S. mutans* in the upper and lower chambers under dual- and multi-species conditions. Data are shown as mean ± SD (n = 6). *p*-values were determined by unpaired t test. *p<0.05, **p<0.01, ***p<0.001.

In the schematic model of microbial transmigration through the mucosal barrier, mucosal integrity is reflected by the trans-epithelial electrical resistance (TEER) value, while microbial transmigration is indicated by the viable cell counts in the lower chamber (**Figure 2A**). Compared to the dual-species condition, the addition of *L. plantarum* increased the TEER value across all sugar conditions, demonstrating its protective effect on mucosal integrity (**Figure 2B**). By numerating the colonies of *S. mutans* that transmigrated into the lower chamber after 24-hour incubation, we found that *L. plantarum* significantly inhibited the transmigration of *S. mutans*, regardless of sugar conditions (**Figure 2C**). By measuring the viable cell counts in both the upper and lower chambers, we observed that *L. plantarum* significantly reduced the viability of *S. mutans* in both chamber (**Figure 2D**).

The expression of *S. mutans* virulence genes *eno* and *gtfC* were significantly downregulated with added *L. plantarum* in 1% sucrose, 1% glucose, or 1% GOS condtions. While, *AptD* in the 1% GOS, *gtfB* in control and 1% GOS conditions, *gtfD* in control and 1% GOS condition, *lacC* across all conditions, and *lacG* in control and 1% glucose conditions were upregulated with the addition of *L. plantarum.* This upregulation might be due to feedback regulation by the surviving *S. mutans* (**Supplementary Figure S1**).

### *L. plantarum* decreases the virulence of *C. albicans*

**Figure 3:**
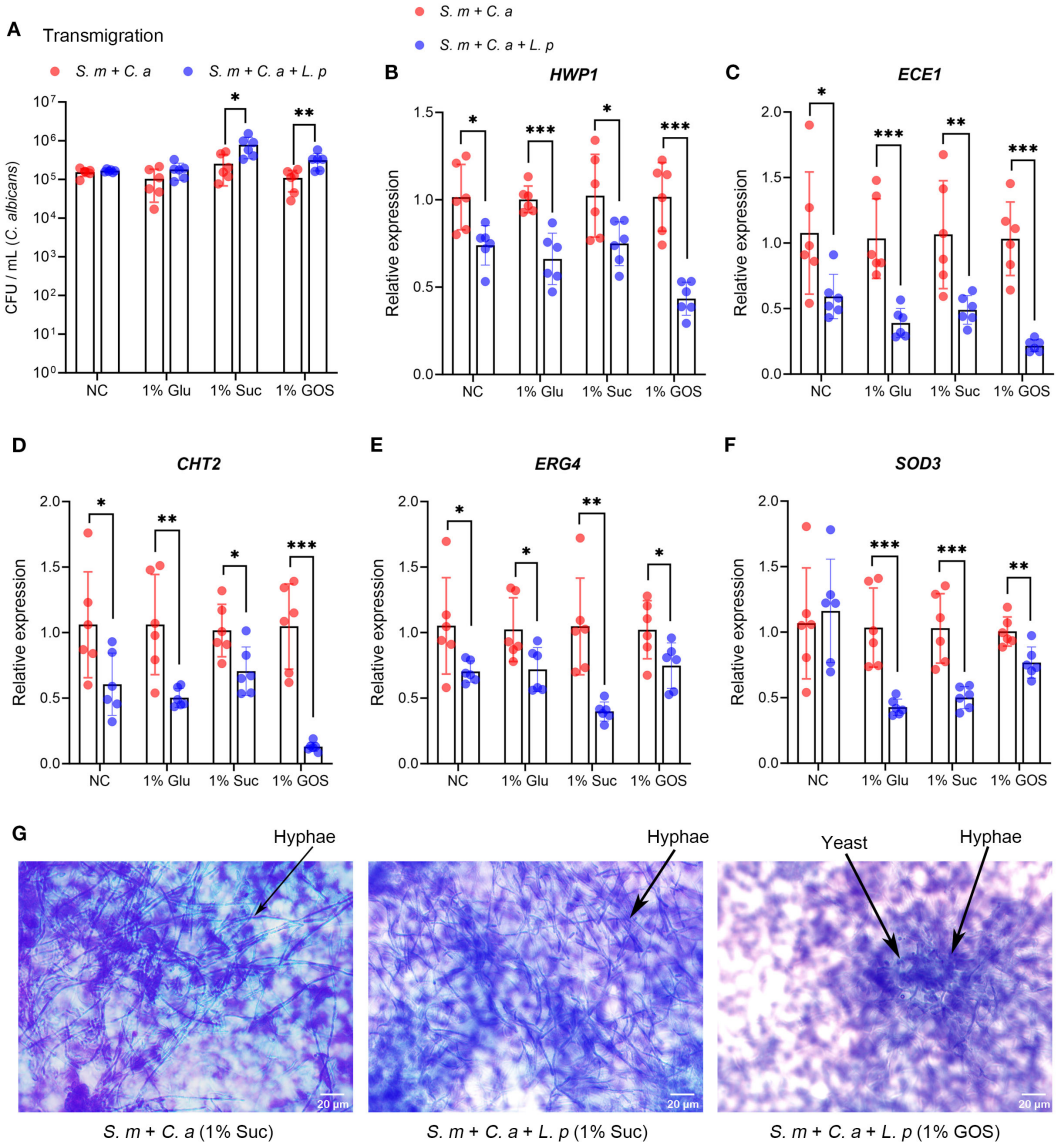
*L. plantarum* decreases virulence gene expression and hyphae formation of *C. albicans*. **(A)**. Viable counts in CFU of C*. albicans* transmigrated from the upper chamber to the lower chamber after 24-hour incubation. **(B-F).** Expression levels of virulence genes in *C. albicans* were measured by qRT-CR after 18 hours of multi-species culture. *L. plantarum* inhibited virulence gene expression of *C. albicans* in sugar conditions. **(G).** The morphology of *C. albicans* in the multi-species mucosal model was assessed using crystal violet staining. *L. plantarum* inhibited hyphae formation of *C. albicans* in 1%GOS culture condition. Data are shown as mean ± SD (n = 6). *p*-values were determined by unpaired t test. *p<0.05, **p<0.01, ***p<0.001.

The migration of *C. albicans* was not significantly inhibited by *L. plantarum* under control and 1% glucose conditions, and it increased under 1% sucrose and 1% GOS conditions (**Figure 3A**). The expression of *C. albicans* virulence genes (*HWP1*, *ECE1*, *CHT2*, *ERG4*, and *SOD3*) was downregulated at 18 hours with the addition of *L. plantarum* (**Figures 3B–F**). Additionally, *C. albicans* hyphae formation was significantly reduced under the 1% GOS condition (**Figure 3G**).

Furthermore, we evaluated the regulatory effects of the prebiotic GOS on the transmigration of *S. mutans* and *C. albicans*. For *S. mutans*, there was no significant difference between 1% GOS and 1% glucose in the dual-species condition, and 1% GOS promoted more *S. mutans* transmigration than 1% sucrose (**Supplementary Figure S2A**, left). In the multi-species condition with *L. plantarum*, the transmigration of *S. mutans* in the GOS group was significantly lower than in the glucose group and showed a decreasing trend compared to the sucrose group (**Supplementary Figure S2A**, right). For *C. albicans*, a downward trend was observed in the 1% GOS group compared with the sucrose group, regardless of whether the condition was dual-species or multi-species (**Supplementary Figure S2B**).

### *L. plantarum* maintains mucosal barrier and reduced cellular immunity

**Figure 4:**
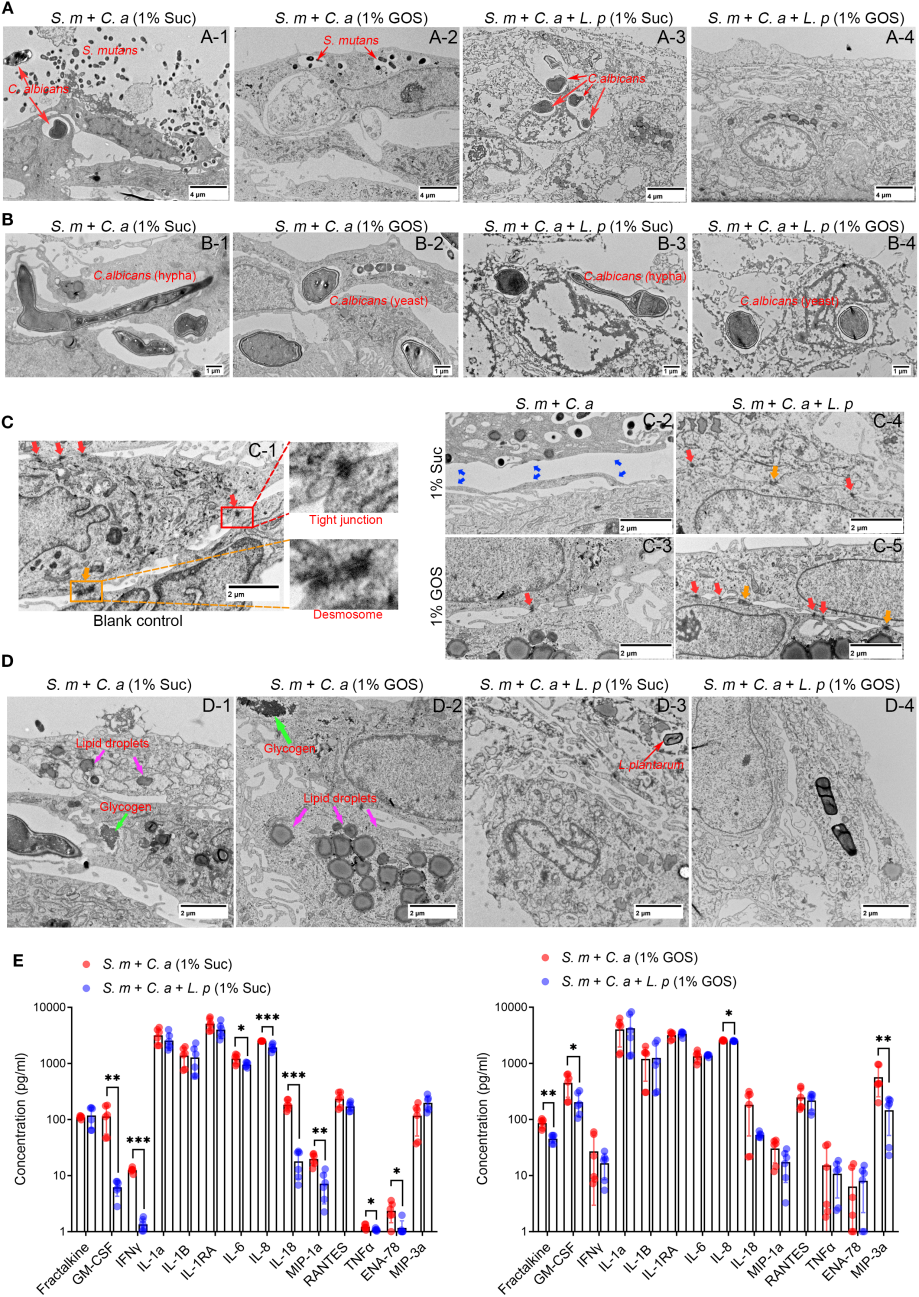
*L. plantarum* maintains mucosal barrier and reduced cellular immunity. **(A)** The morphology of the epithelial cells was assessed using TEM after 12 hours of microbial infection. The addition of *L. plantarum* improved mucosal thickness and integrity, suggesting stronger defense function. **(B)** Effect of *L. plantarum* and prebiotics GOS on the morphology of *C. albicans*. **(C)** Under GOS conditions, the addition of *L. plantarum* enhanced mucosal cell junctions. **(D)** Regulation of lipid droplets and glycogen accumulation in mucosal cells by *L. plantarum* and GOS. **(E)** Concentration of immune markers in the upper chamber of the mucosal model were assessed by Luminex^®^ Multiplex Assays. Under 1% sucrose and 1% GOS conditions, *L. plantarum* inhibited the release of mucosal cell immune markers, suggesting an anti-inflammatory effect. Data are shown as mean ± SD (n = 6). *p-*values were determined by unpaired t test. *p<0.05, **p<0.01, ***p<0.001.

Transmission electron microscopy (TEM) was used to photograph mucosal models under different conditions. In the dual-species model in 1% sucrose condition, *S. mutans* and *C. albicans* damaged the mucosal structure, expanded cell spaces, and invaded cell interiors, leading to cell necrosis (**Figure 4A-1**). In the GOS-containing dual-species condition, barrier damage was slightly reduced, and *C. albicans* invasion was inhibited (**Figure 4A-2**). In the multi-species model with *L. plantarum*, although *C. albicans* still invaded cell interiors in the sucrose-containing condition, the mucosa was significantly thickened, with better integrity than in the dual-species (**Figure 4A-3**). Moreover, in the 1% GOS multi-species condition, the invasion of *S. mutans* and *C. albicans* was notably reduced, and the mucosal barrier was more complete (**Figure 4A-4**), suggesting that *L. plantarum* combined with the prebiotic GOS could better maintain mucosal barrier function. Morphological changes of *C. albicans* observed under electron microscopy were consistent with optical microscopy results. Sucrose contributes to the maintenance of virulent hyphal forms (**Figures 4B-1, 3**), while GOS promotes the transformation of *C. albicans* into yeast forms with lower virulence (**Figures 4B-2, 4**). This further suggests that GOS has an inhibitory effect on the virulence of *C. albicans*.

**Figure 5:**
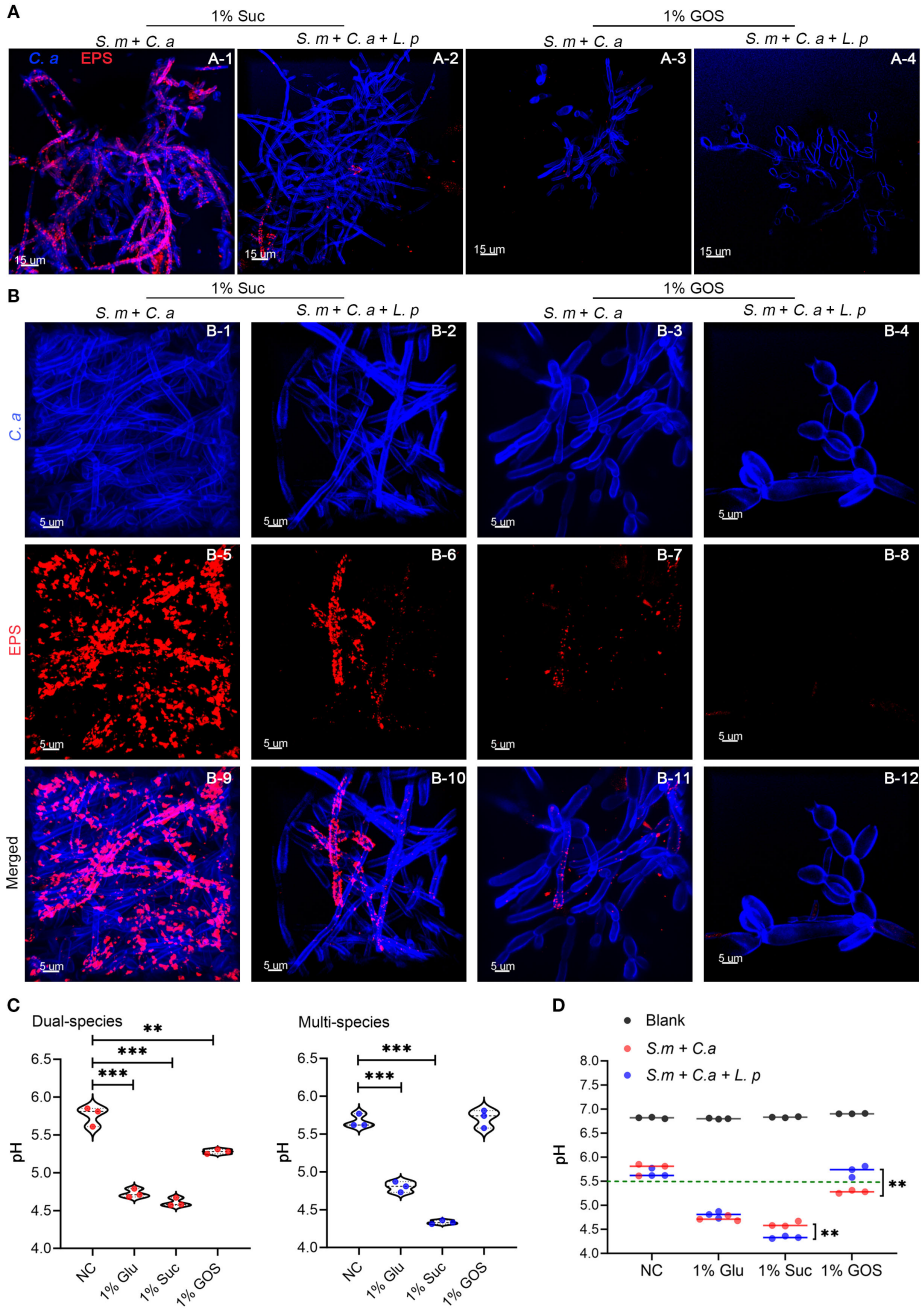
GOS enhanced the ability of *L. plantarum* to inhibit the acid production of *S. mutans*-*C. albicans.* **(A)** *C. albicans* morphology and exopolysaccharide (EPS) accumulation in the mucosal model (12 hours) were observed under sucrose and GOS conditions using confocal laser scanning microscopy (630x). **(B)** *C. albicans* morphology and EPS accumulation were observed at higher magnification (1890x). **(C)** pH levels in dual-species and multi-species models under different sugar conditions. Data are shown as mean ± SD (n = 3). *p*-values were determined by unpaired t test. **p<0.01, ***p<0.001. **(D)** Differences in pH values between dual-species and multi-species models under different sugar conditions. Data are shown as mean ± SD (n = 3). *p*-values were determined by unpaired t test. **p<0.01.

When comparing intercellular connections across different models, we found that in the control group, epithelial cells displayed tight junction (red arrows) and desmosomes (yellow arrows), maintaining cell connection and communication (**Figure 4C-1**). In the dual-species inoculation model, the barrier structure in the sucrose culture was severely damaged, with larger cell gaps, facilitating the invasion and transmigration of pathogenic bacteria (**Figure 4C-2**). In 1% GOS conditions, tight junctions remained, but desmosomes were absent, and cell gaps were still large (**Figure 4C-3**). While, the addition of *L. plantarum* maintained the intercellular junction structure. Both tight junctions and desmosomes were present under sucrose conditions (**Figure 4C-4**), and more were observed under GOS conditions (**Figure 4C-5**), indicating enhanced intercellular connections and improved barrier function. This finding was further supported by cellular immunofluorescence, which showed that occludin and E-cadherin were upregulated in the multi-species models. These proteins are essential for regulating intercellular connections and barrier function in epithelial tissues, helping to maintain tissue integrity and homeostasis (**Supplementary Figure S3**).

We also observed more glycogen accumulation and lipid droplet formation in epithelial cells in the dual-species inoculation model (**Figures 4D-1, 2**). In the multi-species inoculation model, the reduction of lipid droplets and glycogen suggests a weakened inflammatory response, indicating that *L. plantarum* may have anti-inflammatory effects (**Figures 4D-3, 4**). To verify these effects, we examined the levels of immune markers in culture medium using Luminex^®^ Multiplex Assays. The results showed that in 1% sucrose condition, *L. plantarum* significantly downregulated pro-inflammatory factors GM-CSF, IFNγ, IL-6, IL-8, IL-18, MIP-1a, TNFα, and ENA-78 (**Figure 4E**, left). In 1% GOS condition, *L. plantarum* inhibited the release of Fractalkine, GM-CSF, IL-8 and MIP-3a (**Figure 4E**, right). Additionally, *L. plantarum* downregulated the levels of ten pro-inflammatory markers, including Fractalkine, GM-CSF, IFNγ, IL1-RA, IL-8, IL-18, MIP-1a, RANTES, ENA-78 and MIP-3a under glucose conditions (**Supplementary Figure S4A**). In the sugar-free negative control group, the levels of three pro-inflammatory factors—Fractalkine, GM-CSF and MIP-3a—were also found to be downregulated (**Supplementary Figure S4B**).

### GOS enhanced the ability of *L. plantarum* to inhibit the acid production of *S. mutans*-*C. albicans*

**Figure 6:**
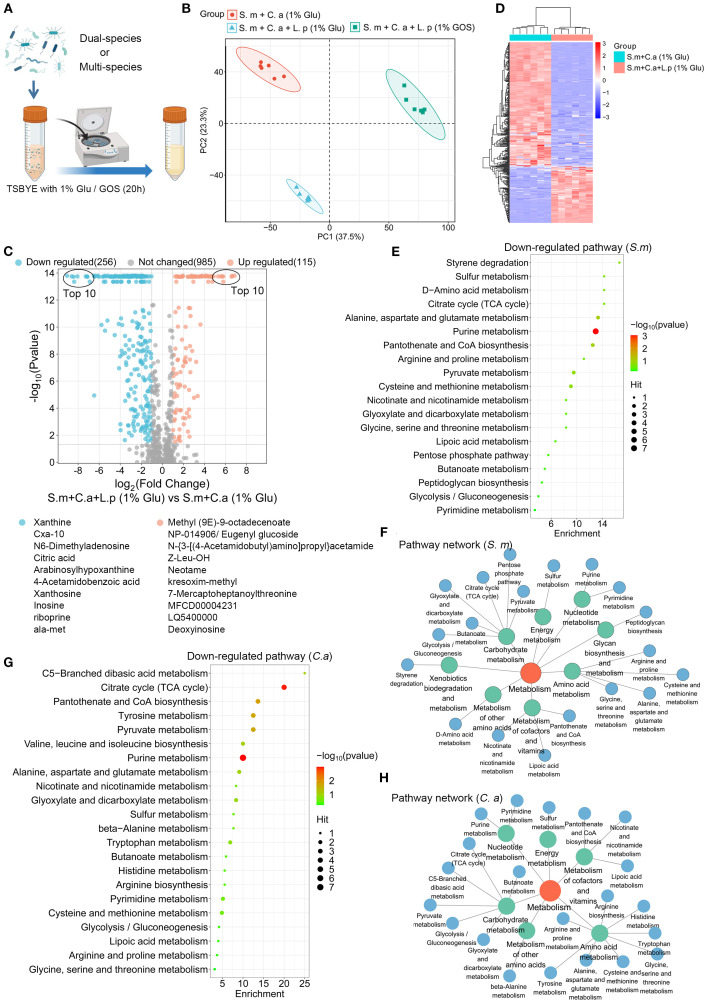
Functional analysis of metabolites regulated by *L. plantarum.* **(A)** Schematic representation of the process of a planktonic model (created with BioRender.com). Dual- and multi-species conditions of *S. mutans* (10^5^ CFU/mL), *C. albicans* (10^3^ CFU/mL), and *L. plantarum* (10^7^ CFU/mL) in 10 mL of TSBYE broth supplemented with 1% GOS or 1% glucose for 20 hours. **(B)** Principal component analysis (PCA) two-dimensional scores plot from the untargeted metabolomics analysis, with each dot representing a biological sample. **(C)** Volcano plot showing 115 up-regulated metabolites (adjusted p < 0.05, log2 FC > 1) and 256 down-regulated metabolites (adjusted p < 0.05, log2 FC < -1) in the multi-species model. **(D)** Clustering heatmap illustrating the classification of metabolites regulated by *L. plantarum* in planktonic models. Rows (metabolites) and columns (samples) are clustered separately, with raw data normalized to Z-scores. The mapping grids are color-coded according to their Z-scores. **(E)** Analysis of down-regulated metabolic pathways using the web-based MetaboAnalyst 6.0, based on *S. mutans* pathway libraries. **(F)** Down-regulated metabolic pathway networks in *S. mutans*. **(G)** Analysis of down-regulated metabolic pathways based on *C. albicans* pathway libraries. **(H)** Down-regulated metabolic pathway networks in *C. albicans*.

**Figure 7:**
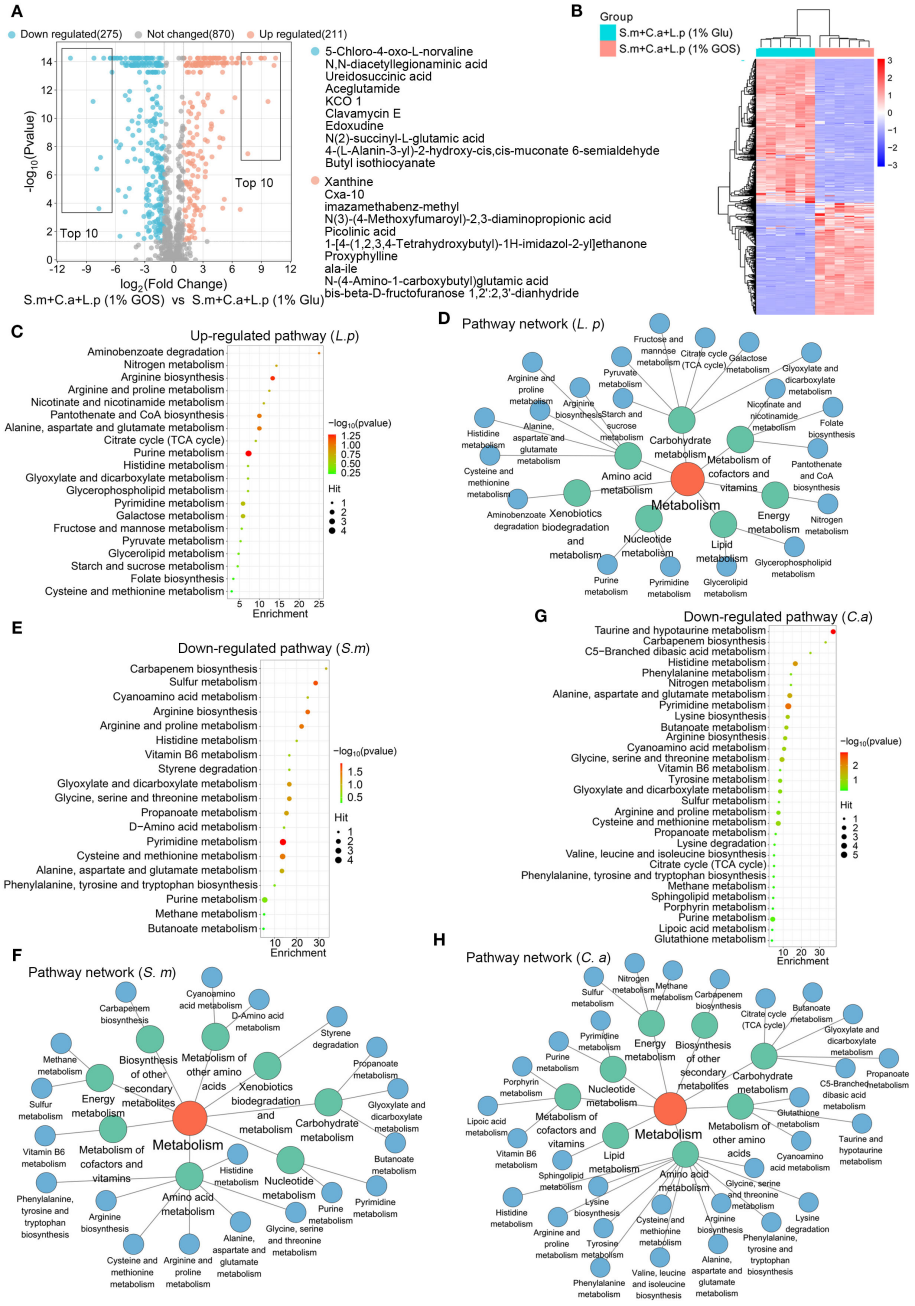
GOS regulated synthesis of functional metabolites in multi-species condition. **(A)** Metabolic volcano plot showing 211 up-regulated (adjusted p < 0.05, log2 FC > 1) and 275 down-regulated (adjusted p < 0.05, log2 FC < -1) metabolites in the multi-microbial model. **(B)** Clustering heatmap illustrating the classification of GOS-regulated metabolites in planktonic models. **(C)** Analysis of up-regulated metabolic pathways, referencing the *L. plantarum* pathway libraries. **(D)** Up-regulated metabolic pathway networks in *L. plantarum*. **(E)** Analysis of down-regulated metabolic pathways referencing *S. mutans* pathway libraries. **(F)** Down-regulated metabolic pathway networks in *S. mutans*. **(G)** Analysis of down-regulated metabolic pathways based on *C. albicans* pathway libraries. **(H)** Down-regulated metabolic pathway networks in *C. albicans*.

Confocal microscopy results demonstrated that the combination of *L. plantarum* and GOS significantly inhibited both EPS accumulation and hyphae formation (**Figure 5A**). In the dual-species model, 1% GOS reduced *C. albicans* hyphae compared to the 1% sucrose condition (**Figure 5B-3**). In the multi-species model, *C. albicans* predominantly remained in yeast form under 1% GOS conditions (**Figure 5B-4**). Moreover, compared to the dual-species model under 1% sucrose conditions (**Figure 5B-5**), both *L. plantarum* addition and the 1% GOS condition significantly reduced EPS production (**Figures 5B-6, 7**). Notably, *L. plantarum* under 1% GOS conditions exhibited the most substantial inhibitory effect on both EPS accumulation and *C. albicans* hyphae formation (**Figure 5B-12**).

The addition of *L. plantarum* significantly inhibited acid production by *S. mutans*-*C. albicans* under 1% GOS conditions. The mean pH of the supernatant in both the dual- and multi-species models was significantly lower under glucose (4.73 vs 4.80, p = 0.0002) and sucrose conditions (4.61 vs 4.33, p < 0.0001) compared to the control group (5.76 vs 5.67). However, under GOS conditions, the pH in both models remained around 5.5 (**Figure 5C**). Notably, in the GOS multi-species group, the pH was higher than in the dual-species group (5.71 vs 5.28, p = 0.0036), approaching the levels observed in the control group (**Figure 5D**).

### Functional analysis of metabolites regulated by *L. plantarum*

The supernatant of a planktonic model was collected for LC-MS/MS untargeted metabolomics analysis to evaluate the effects of *L. plantarum* on the metabolism of *S. mutans* and *C. albicans* (**Figure 6A**). The clustering of samples in PCA plots indicated distinct global metabolomic profiles between groups (**Figure 6B**). We found that 371 metabolites were significantly altered with the addition of *L. plantarum*, with 256 down-regulated metabolites and 115 up-regulated metabolites, with the top ten most significantly up-regulated and down-regulated metabolites listed (**Figure 6C**). A clustering heatmap revealed clear differences in altered metabolite profiles between dual-species and multi-species planktonic models (**Figure 6D**).

Pathway analysis of the significantly down-regulated metabolites (p-value < 0.05, log2 (Fold Change) < -1) was conducted based on *S. mutans* and *C. albicans* pathway libraries. *L. plantarum* significantly down-regulates the metabolic pathways of *S. mutans* and *C. albicans* that are crucial for energy and biomolecule production. For *S. mutans*, 19 metabolic pathways were identified, with purine metabolism being the most down-regulated (**Figure 6E**). These pathways were primarily enriched in carbohydrate metabolism, amino acid metabolism, and the metabolism of cofactors and vitamins (**Figure 6F**). For *C. albicans*, 22 metabolic pathways were identified, with purine metabolism and the citrate cycle being the most significantly down-regulated (**Figure 6G**). Similar enrichments were observed in *C. albicans* (**Figure 6H**).

### GOS regulated synthesis of functional metabolites in multi-species condition

We also examined the metabolic regulatory effects of GOS on *L. plantarum, S. mutans* and *C. albicans* in multispecies planktonic model. Volcano plots indicated that under GOS conditions, 275 metabolites were down-regulated, and 211 were up-regulated compared to glucose conditions (**Figure 7A**). Heatmap results showed significant differences in metabolite profiles between GOS and glucose conditions in a multi-species planktonic model (**Figure 7B**). Pathway analysis of the up-regulated metabolites was performed using the *L. plantarum* pathway library. Twenty metabolic pathways were identified, with significant up-regulation in purine metabolism, alanine, aspartate and glutamate metabolism, pantothenate and CoA biosynthesis, and arginine biosynthesis (**Figure 7C**). These pathways were mainly enriched in carbohydrate metabolism, amino acid metabolism, and the metabolism of cofactors and vitamins (**Figure 7D**).

Pathway analysis of the down-regulated metabolites was performed using *S. mutans* and *C. albicans* pathway libraries. For *S. mutans*, 19 metabolic pathways were identified, with pyrimidine metabolism, cysteine and methionine metabolism, and sulfur metabolism being the most significantly down-regulated (**Figure 7E**). These pathways were mainly enriched in amino acid metabolism, carbohydrate metabolism, and the metabolism of cofactors and vitamins (**Figure 7F**). For *C. albicans*, 30 metabolic pathways were identified, with significant down-regulation observed in pyrimidine metabolism and taurine and hypotaurine metabolism (**Figure 7G**). Here, the pathways related to amino acid metabolism, carbohydrate metabolism, metabolism of other amino acids, metabolism of cofactors and vitamins, energy metabolism, and cofactors and vitamins metabolism were frequently enriched (**Figure 7H**).

### Functional regulation of metabolic landscape by *L. plantarum* under GOS conditions

**Figure 8:**
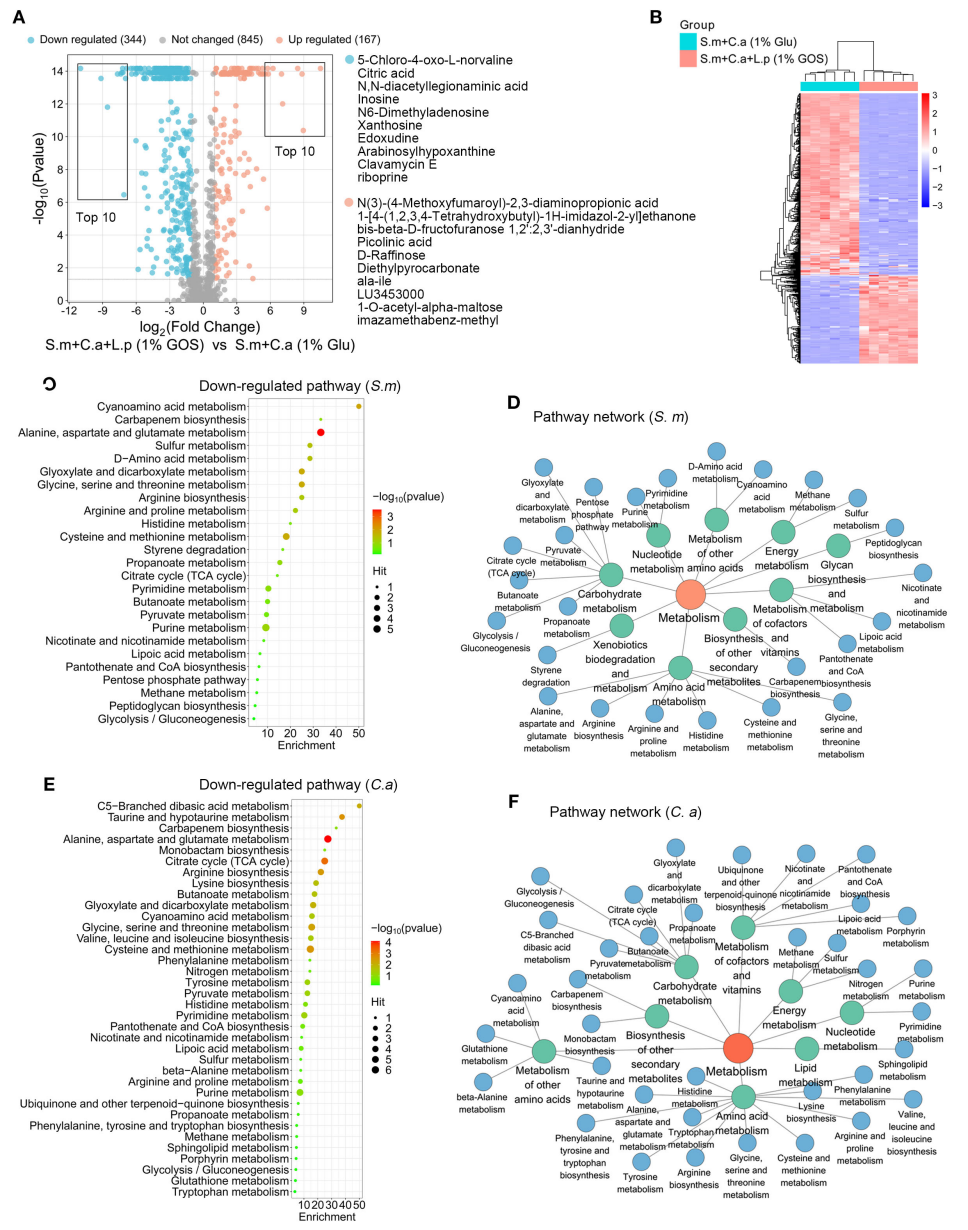
Functional regulation of metabolic landscape by *L. plantarum* under GOS conditions. **(A)** Metabolic volcano plot showing 167 up-regulated (adjusted p < 0.05, log2 FC > 1) and 344 down-regulated metabolites (adjusted p < 0.05, log2 FC < -1) in the multi-microbial model. **(B)** Clustering heatmap illustrating the classification of metabolites regulated by *L. plantarum* and GOS in planktonic models. **(C)** Analysis of down-regulated metabolic pathways referencing *S. mutans* pathway libraries. **(D)** Down-regulated metabolic pathway networks in *S. mutans*. **(E)** Analysis of down-regulated metabolic pathways based on *C. albicans* pathway libraries. **(F)** Down-regulated metabolic pathway networks in *C. albicans*.

The volcano plot showed that 371 metabolites changed significantly with the addition of *L. plantarum* under GOS condition, with 344 metabolites down-regulated and 167 up-regulated (**Figure 8A**). The heatmap revealed notable differences in altered metabolite profiles between dual-species (1% glucose) and multi-species (1% GOS) conditions in planktonic models (**Figure 8B**).

For down-regulated metabolites, pathway analysis was conducted using the *S. mutans* and *C. albicans* pathway libraries. For *S. mutans*, 25 metabolic pathways were identified, with purine metabolism being the most down-regulated (**Figure 8C**). Similar to *L. plantarum* under glucose conditions, these pathways were mainly enriched in carbohydrate metabolism, amino acid metabolism, and the metabolism of cofactors and vitamins. However, a notable difference was the presence of pathways related to xenobiotic biodegradation and metabolism. Microorganisms, including bacteria and fungi, play a crucial role in the biodegradation of xenobiotics, utilizing specific enzymes to metabolize these compounds (**Figure 8D**).

For *C. albicans*, 36 metabolic pathways were identified, with significant down-regulation in alanine, aspartate and glutamate metabolism, cysteine and methionine metabolism, glycine, serine and threonine metabolism, the citrate cycle, and taurine and hypotaurine metabolism (**Figure 8E**). Here, pathways related to amino acid metabolism, carbohydrate metabolism, metabolism of other amino acids, metabolism of cofactors and vitamins, and energy metabolism were frequently enriched (**Figure 8F**).

## Discussion

### Highlights

This study demonstrated inhibitory effects of *L. plantarum* and the prebiotic GOS on *S. mutans* and *C. albicans* in mucosal models. Our findings indicate that under GOS conditions, *L. plantarum* induces anti-inflammatory effects in mucosal infection models and reshapes the metabolic landscape of planktonic models, leading to reduced viability and virulence of both *S. mutans* and *C. albicans*, thereby maintaining mucosal integrity.

### The inhibitory effect of *L. plantarum* and GOS on *S. mutans*-*C. albicans*

The interaction between *S. mutans* and *C. albicans* in saliva and biofilm environments plays a critical role in ECC and other oral mucosal infections. *S. mutans* synthesizes EPS, promoting the formation of dense biofilms. Within these biofilms, *S. mutans* ferments sugars, producing lactic acid as a byproduct. EPS further enhances the retention of sugar substrates and acid, leading to a localized pH reduction. The risk of caries increases when saliva pH drops to 5.5 or lower. *C. albicans* is a polymorphic species capable of transforming between single-celled yeast and multicellular hyphae to adapt to environmental changes (Liang et al., 2024; Moffa et al., 2015). Additionally, the expression of its virulence genes varies under specific environmental stress, influencing its pathogenicity (Verma-Gaur and Traven, 2016; Muñoz et al., 2018). In this study, we found that *L. plantarum* significantly reduced the adherence of *S. mutans* and *C. albicans* to the mucosal barrier, thereby inhibiting EPS accumulation. Moreover, *L. plantarum* directly inhibits the viability and transmigration of *S. mutans*. It also reduces the virulence and pathogenicity *C. albicans* by suppressing hyphae formation and downregulating virulence gene expression. Furthermore, the GOS condition enhanced this inhibitory effect more effectively than glucose and sucrose conditions.

### The protective effect of *L. plantarum* and GOS on mucosal barrier

The oral mucosa is crucial for barrier protection by maintaining thickness, integrity, cellular connectivity, and providing immune defense against pathogen invasion (Muñoz et al., 2018; Ptasiewicz et al., 2022; Gaffen and Moutsopoulos, 2020). In this study, we identified that GOS enhanced the protective effects of *L. plantarum* on mucosal integrity. In the group inoculated with *L. plantarum* under GOS conditions, mucosal thickness and intercellular connections increased, ensuring normal barrier function. These findings illustrate the potential of *L. plantarum*, especially in conjunction with GOS, to modulate microbial virulence and maintain mucosal barrier integrity, indicating promising therapeutic avenues for oral health interventions.

### Regulation of *L. plantarum* and GOS on metabolic landscape

Untargeted metabolomics is a valuable tool for discovering products of cellular biochemical reactions in various diseases (Ramos et al., 2022; Chen et al., 2022; Crestani et al., 2020; Yang et al., 2022; Niziol et al., 2023). Our study showed that *L. plantarum* can inhibit pathogenic bacteria by regulating the metabolic microenvironment. We utilized LC-MS/MS to identify thousands of metabolites. Among the ten most significantly up-regulated metabolites by *L. plantarum*, methyl (9E)-9-octadecenoate, eugenyl glucoside, neotame and kresoxim-methyl altered the diversity of the gut microbiome and likely exhibited antimicrobial activities (Chi et al., 2018; Zhao et al., 2010; Rozman et al., 2017; Filippou et al., 2016). Conversely, the most down-regulated metabolites, including xanthine, CXA-10, citric acid, arabinosylhypoxanthine, inosine, and riboprine, serve as nutrient sources that promote microbial proliferation (Martin-Gallausiaux et al., 2021; Trefely et al., 2020). For GOS, the up-regulated metabolites, such as cxa-10, proxyphylline, and N-(4-Amino-1-carboxybutyl)glutamic acid have bactericidal properties (Moya et al., 2010; Ajayeoba et al., 2019; Fazly Bazzaz et al., 2016). Meanwhile, down-regulated metabolites, including ureidosuccinic acid, aceglutamide, and 4-(L-Alanin-3-yl)-2-hydroxy-cis, cis-muconate 6-semialdehyde, can enhance bacterial and fungal growth through protein synthesis and other metabolic pathways (Zeng et al., 2024; Zhou et al., 2023; Martino et al., 2018; Cai et al., 2007). Among the top ten metabolites up-regulated by *L. plantarum* and GOS, N(3)-(4-Methoxyfumaroyl)-2,3-diaminopropionic acid, picolinic acid and pyrogallol are recognized for their antimicrobial properties (Otite et al., 2024; Tian et al., 2023). In contrast, the top down-regulated metabolites included xanthosine and inosine, both nucleosides that serve as nutrient sources capable of stimulating microbial growth (McKeague et al., 2016; Li et al., 2021). Furthermore, our analysis of these regulated pathways elucidates the metabolic interactions among *L. plantarum*, *S. mutans*, and *C. albicans*, indicating that *L. plantarum* significantly alters the metabolic landscape of these pathogens, especially under GOS conditions. The down-regulation of critical nutrient metabolic pathways in *S. mutans* and *C. albicans* suggests a competitive suppression mechanism initiated by *L. plantarum*, potentially impairing their energy production and virulence. Conversely, the up-regulation of these nutrient or energy metabolic pathways in *L. plantarum* emphasizes its adaptability and resilience, enhancing its survival and functionality within the oral cavity.

### The anti-inflammatory effects of *L. plantarum*

Immunometabolism is a multidisciplinary area of immunology research that intricately links metabolism and immunology. It has emerged as a central mechanism in adaptive and innate immune regulation (Makowski et al., 2020). Glycogen and lipid droplets serve as energy reserves in metabolism and also have immunomodulatory functions, increasing during infection and inflammation to regulate the inflammatory response (Marschallinger et al., 2020; Sadiku et al., 2021). Transmission electron microscopy revealed that *L. plantarum* reduces the accumulation of lipid droplets and glycogen, which are indicators of an enhanced cell autoinflammatory response. This suggests that *L. plantarum* may reduce the autoimmunity of mucosal cells. Pro-inflammatory markers play key roles in immune responses. Luminex results confirmed a significant reduction in immune marker release in the *L. plantarum* added group. Generally, these markers orchestrate inflammatory and immune responses, with TNFα stimulating cytokines like IL-6 and chemokines such as IL-8 and MIP-1a (Lee and Moon, 2023; Abbott et al., 2021; Beringer and Miossec, 2019). IL-6 enhances acute-phase protein production, while IL-18, in synergy with IL-12, promotes IFNγ production to strengthen Th1 responses (Chen et al., 2021; Ngwa et al., 2022; Yasuda et al., 2019; Wawrocki et al., 2016). GM-CSF facilitates immune cell maturation, and fractalkine recruits immune cells, amplifying cytokine effects (Subbotin, 2014; Yang et al., 2024; Kumar et al., 2022). The observed reduction in these pro-inflammatory markers suggests that *L. plantarum* may help regulate inflammation and maintain normal cell function.

## Conclusions

In conclusion, our findings emphasize the antimicrobial properties of probiotics against oral pathogens. Specifically, GOS has a synergistic effect in enhancing the viability of *L. plantarum* to inhibit *S. mutans* and in converting the virulent hyphae form of *C. albicans* to a less pathogenic yeast form. Compared to previous studies that only focused solely on single drugs, this research supplements the understanding of probiotic interventions in oral health, offering a novel approach to managing the oral microbiome. By leveraging the natural dynamics within the oral microbiome, innovative probiotic therapies could emerge to mitigate the impact of oral pathogens, improve mucosal health, and reduce the incidence of dental diseases.

## Limitations

Despite the promising findings, this study has limitations. The *in vitro* nature of our experiments may not fully replicate the complex dynamics of the oral microbiome *in vivo*. Additionally, while we identified several metabolic pathways affected by *L. plantarum*, future studies should employ more comprehensive metabolomic approaches to elucidate their implications for oral health. Future research should also focus on the specific molecular interactions and regulatory networks involved in the metabolic and immune shifts induced by *L. plantarum*. Understanding these interactions could provide deeper insights into its therapeutic potential.

## Data Availability

All data generated or analyzed during this study are included in this article. Further inquiries can be directed to the corresponding author.
