# Supplementary material for: Metabolic modulation and multi-species interaction: Lactiplantibacillus plantarum’s impact on Streptococcus mutans-Candida albicans in a mucosal model
Source: Front Cell Infect Microbiol. 2025 Oct 8;15:1652490. doi: 10.3389/fcimb.2025.1652490 (PMC12540473; doi:10.3389/fcimb.2025.1652490)
Supplement: Supplementary file 1 [file DataSheet1.pdf]

## Supplementary figures

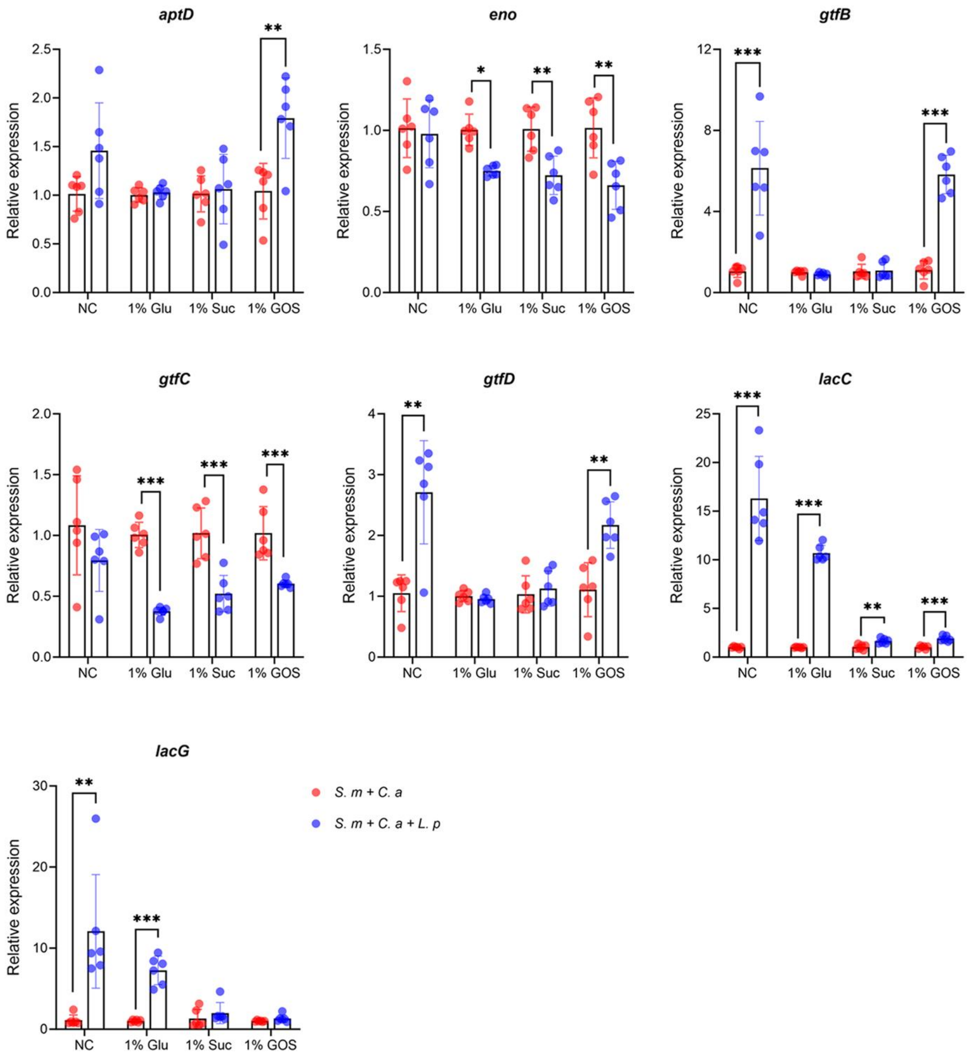


**Fig. S1: Inhibitory effect of *L. plantarum* on *S. mutans* virulence Gene Expression in a multi-species mucosal model.**

The expression of virulence genes in *S. mutans* was measured by qRT-PCR after 18 hours of multi-species culture. *L. plantarum* inhibited mRNA expression of *eno* and *gtfC*, while weakly regulating or even enhancing other *S. mutans* virulence genes. This may result from the feedback effect of surviving pathogens after *L. plantarum* significantly reduces *S. mutans* populations. Data are shown as mean ± SD (n = 6). *p*-values were determined by unpaired t test. * $p<0.05$, ** $p<0.01$, *** $p<0.001$.

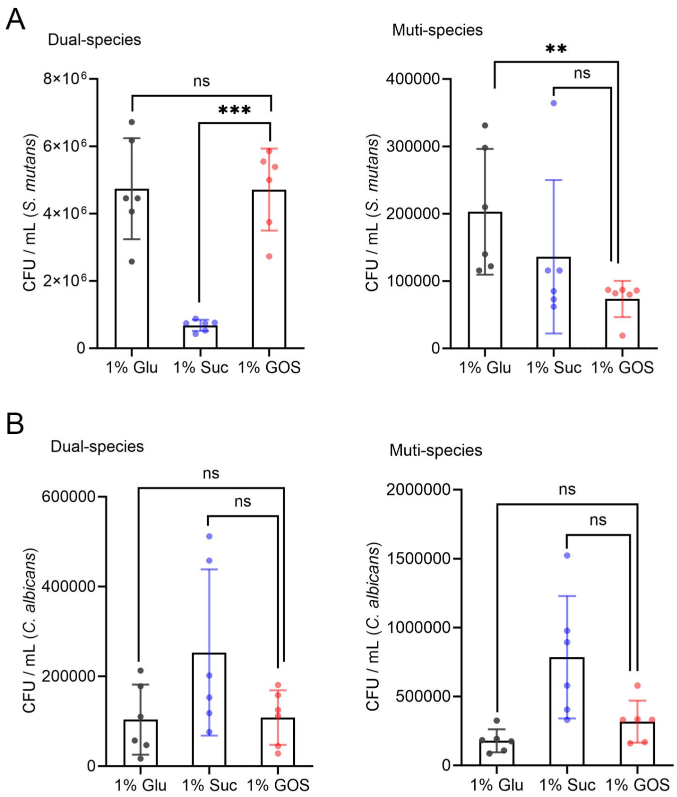


**Fig. S2: Inhibitory effect of *L. plantarum* and GOS on the transmigration of *S. mutans* and *C. albicans*.**

**A.** Viable counts of *S. mutans* in the lower chambers under dual- and multi-species conditions. **B.** Viable counts of *C. albicans* in the lower chambers under dual- and multi-species conditions. Data are shown as mean ± SD (n = 6). p-values were determined by one-way ANOVA. * $p<0.05$, ** $p<0.01$, *** $p<0.001$; ns, not significant.

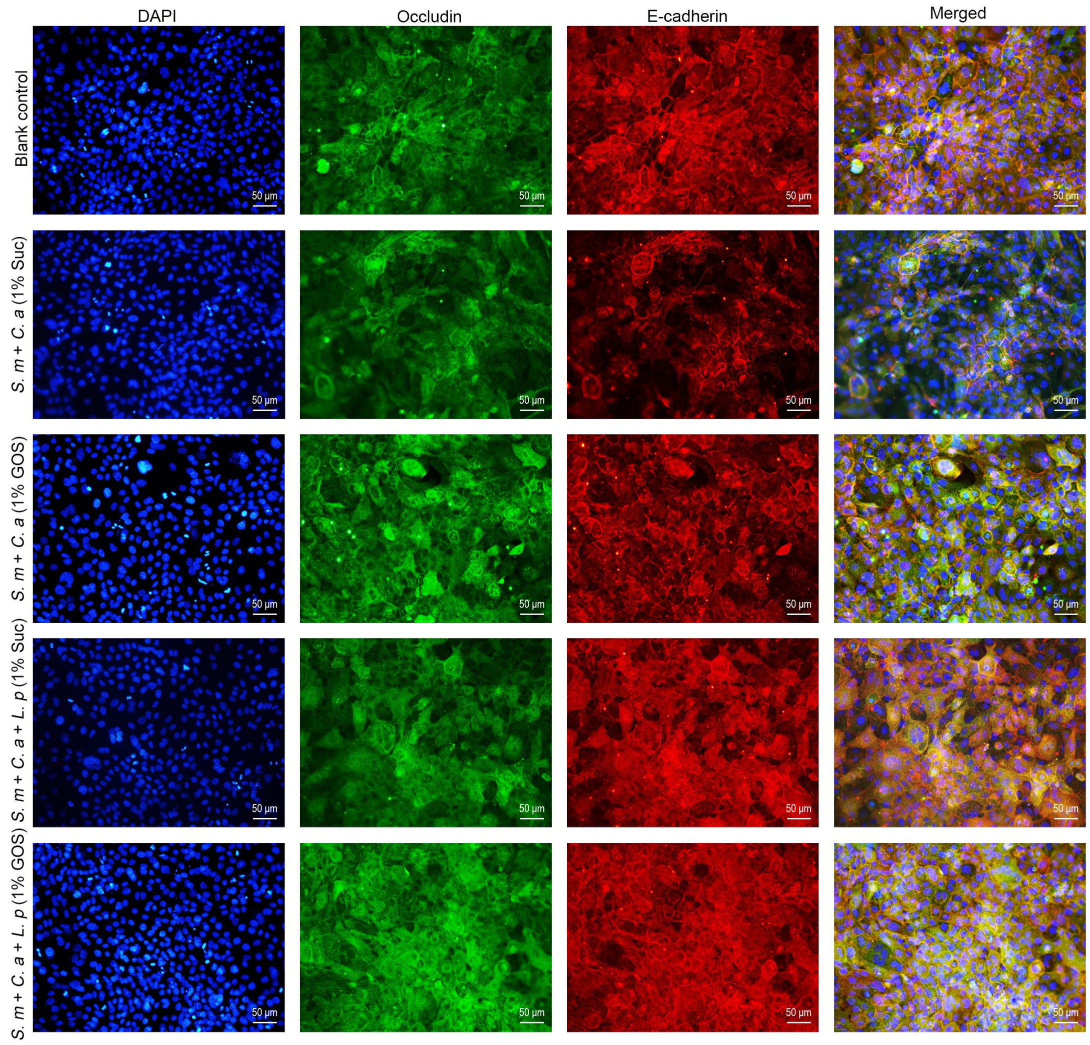


**Fig. S3: *L. plantarum* and GOS increased the expression of junction Occludin and E-cadherin.**

Protein expressions of Occludin and E-cadherin were detected using immunostaining. *L. plantarum* and GOS enhance mucosal integrity by significantly increasing tight junction and adherent junction proteins. DAPI, a bright blue fluorescent molecule that binds reversibly to double-stranded DNA, was used to visualize chromosomes and nuclear stain.

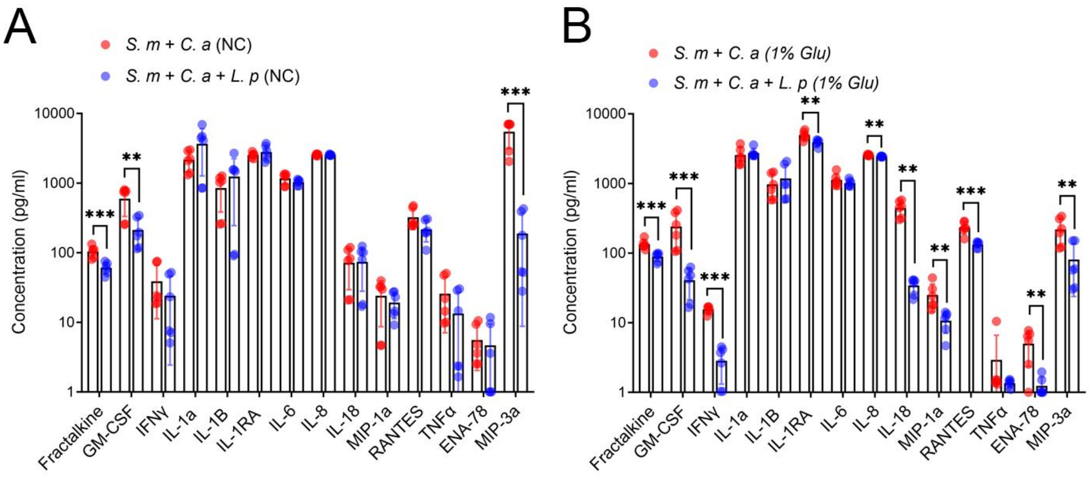


**Fig. S4: Inhibitory effect of *L. plantarum on* cellular immunity.**

**A**. Inhibitory effect of *L. plantarum* on mucosal cell immune markers under negative control condition. **B.** Inhibitory effect of *L. plantarum* on mucosal cell immune markers under glucose condition. Immune markers were assessed using Luminex® Multiplex Assays. Data are shown as mean ± SD (n = 6). *p*-values were determined by unpaired t test. * p<0.05, ** p<0.01, *** p<0.001.

**Supplementary tables**

**Table S1. PCR primers, related to experimental procedures**

| Gene | Forward Primer Sequence (5'-3') | Reverse Primer Sequence (5'-3') | Amplicon Size (bp) |
|---|---|---|---|
| *HWP1* | TGGTGCTATTACTATTCCGG | CAATAATAGCAGCACCGAAG | 182 |
| *ECE1* | GCTGGTATCATTGCTGATAT | TTCGATGGATTGTTGAACAC | 168 |
| *CHT2* | TTCGATGGATTGTTGAACAC | GCAGAAGAAGATGGGGCAACAC | 111 |
| *ERG4* | TCAAATGTGCCAATGGTTCT | AGCCCAAGTCAATGTTTGAA | 101 |
| *SOD3* | CAGTATGGGTCTGTTTCAAACCTTA | GATATTGCAAGTAGTACGCATGTTC | 211 |
| *ACT1* | TGCTCCAGAAGAACACCCA | CACCTGAATCCAAAACAATACCAGT | 182 |
| *aptD* | TGTTGATGGTCTGGGTGAAA | TTTGACGGTCTCCGATAACC | 176 |
| *eno* | CAGCGTCTTCAGTTCCATCA | TCACTCAGATGCTCCAATCG | 194 |
| *gtfB* | AGCAATGCAGCCATCTACAAAT | ACGAACTTTGCCGTTATTGTCA | 96 |
| *gtfC* | CTCAACCAACCGCCACTGTT | GGTTTAACGTCAAAATTAGCTGTATTAGC | 91 |
| *gtfD* | CACAGGCAAAAGCTGAATTAACA | GAATGGCCGCTAAGTCAACAG | 81 |
| *lacC* | GCTGGAATTACATCGGCTCTTGC | CCTCCGCTACCTCAATTTGTTGG | 157 |
| *lacG* | ATTGGATGCGTGCTTTTGATGG | CGACCGACACCCTTAATCTGG | 94 |
| *gyrA* | CCAAGAATCTGCTGTCCG | TTGCGACTATCTGCTATGTG | 111 |

**Table S2. Antibodies for immunostaining**

| Antibody | Vendor | Cat. No. | Dilution |
|---|---|---|---|
| Rabbit Anti-Occludin IgG | Invitrogen | 71-1500 | 1:100 |
| Mouse Anti-E-cadherin IgG | Invitrogen | 13-1700 | 1:1000 |
| FITC-Goat Anti-Rabbit IgG(H+L) | Invitrogen | 65-6111 | 1:2000 |
| Alexa Fluor-Donkey Anti-Mouse IgG(H+L) | Invitrogen | A-31570 | 1:500 |
